# Bipolar sternocleidomastoid partial resection in pediatric patients case report: Technique and early outcomes

**DOI:** 10.1097/MD.0000000000040935

**Published:** 2024-12-27

**Authors:** Kyle K. Obana, Annika Y. Myers, James T. Bennett, Ali A. Siddiqui, Bensen B. Fan, Lindsay M. Andras, Tishya A.L. Wren, David L. Skaggs

**Affiliations:** a Department of Orthopedic Surgery, Columbia University, New York, NY; b Jackie and Gene Autry Orthopedic Center, Children’s Hospital Los Angeles, Los Angeles, CA; c Department of Orthopedic Surgery and Sports Medicine, Children’s Hospital of the King’s Daughters, Norfolk, VA; d Department of Orthopedics, University of Florida College of Medicine – Jacksonville, Jacksonville, FL; e Children Center for Orthopedics, Orlando Health Arnold Palmer Hospital, Orlando, FL; f Department of Orthopedics, Cedars-Sina Medical Center, Los Angeles, CA.

**Keywords:** bipolar, case report, partial resection, release, sternocleidomastoid

## Abstract

**Rationale:**

Congenital muscular torticollis (CMT) that is refractory to conservative therapy is often treated with unipolar or bipolar sternocleidomastoid (SCM) release. Prior texts and descriptions warn of overcorrection or defects, but these have not been encountered in our practice. However, recurrence and undercorrection have been observed, prompting us to transition to a bipolar release with partial resection of SCM. We will review the outcomes of this modified approach.

**Patient concerns:**

Nine patients (6 males and 3 females) failed a trial of physical therapy and all conservative measures.

**Diagnoses:**

All patients were diagnosed with CMT refractory to conservative therapy.

**Interventions:**

Nine patients were treated with unipolar SCM (6 right and 3 left). The mean age at the time of surgery was 9.3±3.4 years.

**Outcomes:**

There were no surgical complications. No patients required subsequent revision surgery. One patient had prior surgery on the SCM.

At final follow-up, 8 (88.9%) patients had a neutral head position. Rotation in the direction of the release significantly improved from preoperative to final follow-up (from 59.7±26.5 to 85.6±7.3 degrees, *P* = .01). Rotation in the opposite direction (away from the release) was maintained from preoperative to final follow-up (83.8±11.1 vs. 85.6±8.8 degrees, *P* = .71). No cases of overcorrection or defect in the area of the resection were observed.

**Lessons:**

Bipolar release with partial resection may be a promising form of treatment for CMT as head position was improved without overcorrection, defects, or other complications.

## 1. Introduction

Congenital muscular torticollis (CMT) is an uncommon condition (incidence 0.3%–2%) associated with a packaging deformity that causes fibrosis and shortening of the sternocleidomastoid (SCM) muscle.^[1,2]^ The pathophysiology is not completely understood, but it is believed that it may be due to intra-uterine crowding, compartment syndrome, fibrosis from peripartum bleeding, or venous outflow obstruction.^[2]^ The result is a contraction of the SCM muscle causing rotation of the chin to the contralateral side and head tilt toward the ipsilateral side.^[3–5]^ Various forms of noninvasive treatment are utilized, including observation, physical therapy, and stretching.^[3,6,7]^ Previous findings suggest minimal benefit of noninvasive procedures once a child is 1 year old.^[6,8,9]^ Hollier et al^[10]^ found that 64% of pediatric patients over 8 years of age responded favorably to physical therapy without subsequent surgery, although 27% of these patients exhibited residual head tilt.

CMT that is refractory to conservative therapy is often treated with unipolar or bipolar SCM release to achieve desired cosmesis and prevent deformities such as facial asymmetry, plagiocephaly, and scoliosis.^[11]^ In particular, unipolar release, bipolar release, and bipolar release with z-plasty of the SCM muscle have been described as common techniques when treating CMT.^[6]^ Currently, the majority of literature analyzes outcomes of bipolar release of the SCM in adult patients showing improved head positioning and cervical range of motion.^[1,5,12–14]^

There are few studies that have investigated the outcomes of bipolar release of the SCM in the pediatric population.^[7,10,15,16]^ In addition, the majority of these studies included patients 18 years of age or older as well as patients undergoing unipolar release.^[7,16]^ Hollier et al^[10]^ investigated the outcomes of patients undergoing physical therapy or bipolar release of the SCM. This cohort consisted of only 4 patients undergoing surgery, one of which was 18 years of age, and they reported a 50% reoperation rate.^[10]^ One study by Ferkel et al^[6]^ showed that bipolar release with z-plasty yielded better outcomes with fewer reoperations compared to other surgical procedures in pediatric patients; however of the 12 patients that had a bipolar release with z-plasty 1 had residual head tilt, 1 had prominence of the scar, and 4 had limited head rotation.

Prior texts and descriptions warn of overcorrection or defects, but these have not been encountered in our practice. However, recurrence and under-correction have been observed, prompting us to transition to a bipolar release with partial resection of SCM. The current case series introduces a new technique to address the high rate of reoperation with bipolar SCM release (up to 50%), which includes resection of part of the muscle.^[10]^ This paper describes the technique and our initial results.

## 2. Procedure

This is a soft-tissue procedure so no fluoroscopy is required. The patient is positioned supine on the operating room table and general anesthesia is administered with endotracheal intubation. We find it helpful to have the endotracheal tube secured to the opposite side of the mouth so that it is out of the way. The patient is then levitated and positioned with a gel roll placed longitudinally under the operative scapula to allow for extension of the head and rotation to the contralateral side to put the operative SCM on stretch. Drape as wide as possible with 10/10 drapes to allow access to the mastoid process as well as the sternum and clavicle. We find it helpful to tape up the earlobe to allow full access to the mastoid. The patient receives perioperative antibiotics. The patient is prepped and draped in normal orthopedic fashion with blue towels, ioban, and split drapes.

**Figure 1.**
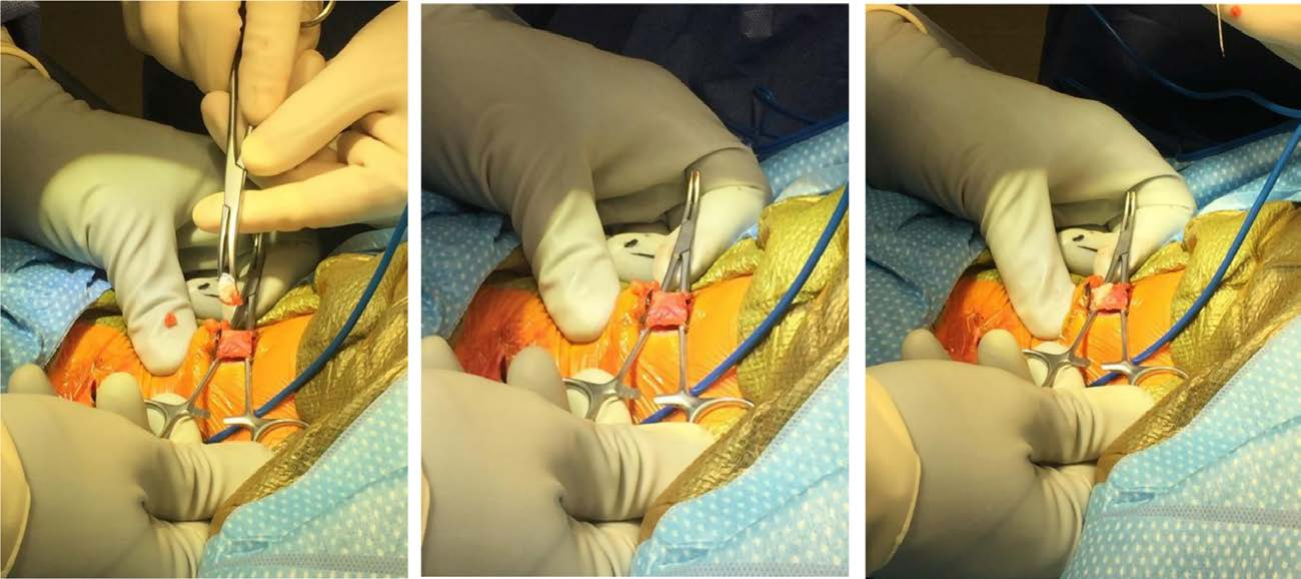
Use of hemostat to bring the SCM muscle out of the wound while protecting it from the skin. To the left is the head of the patient, to the right are the lower extremities.

**Figure 2.**
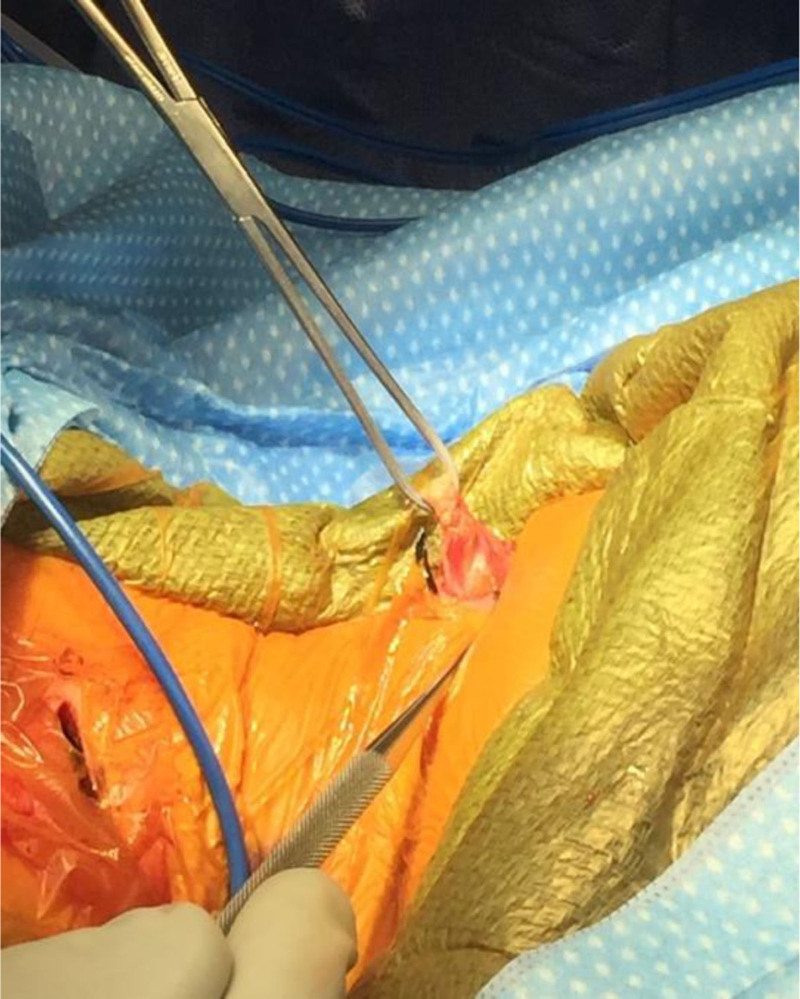
Muscle release 1 cm from attachment using a bovie.

The incision is marked out by palpating the origin of the sternal and clavicular heads of the muscle as well as the insertion of the muscle at the mastoid. A 2 cm transverse incision is made in line with Langers lines with a 15-blade scalpel around 1 cm above the clavicle, and the dissection is completed down to the level of the SCM through the platysma with Bovie electrocautery. A separate incision is made in the width of the muscle just below the mastoid. For these smaller incisions, a needle tip bovie bent at a 45° angle is helpful. Palpate the tight cords of the muscle and use a freer or Ragnell retractors to provide visualization of the muscle. Bluntly perform extra-fascial dissection around the muscle with a hemostat circumferentially and pass a 90° forceps around the muscle ensuring you are directly around the muscle. Use a peanut to remove any fat from the muscle. It is helpful to slide a hemostat or Crile retractor under the muscle so that the width of the instrument helps bring the muscle out of the wound while also protecting it from the skin (Fig. 1). It is wise to have a retractor or hemostat circumferentially around the muscle at both incisions prior to any release as it is more easily identified while under tension. From the incision just below the mastoid, the muscle is then released 1 cm from its attachment with a bovie (Fig. 2). It is important to do this slowly, about 1 mm at a time, to ensure that only the muscle is released. Beware of the spinal accessory nerve that innervates the muscle. An Allis clamp is placed on the remaining muscle to allow slight tension on the muscle and making sure that the muscle is free around distal to where it was transected and then a 1 cm segment is resected. The muscle at the origin just above the clavicle is transected and if needed an additional segment can be resected there as well. The muscle is then palpated to ensure there are no remaining bands, which can be resected in a similar manner. It is imperative that there are no remaining bands which are sources of tension.

The incisions are copiously irrigated with sterile saline and closed in layers using 2-0 vicryl buried sutures for the platysma, followed by 4-0 monocryl running subcuticular suture, and dermabond. We have found that further dressings are not necessary and may even lead to skin tension injuries. The patient is placed in a pinless halo while still intubated. The head should be placed in a position of overcorrection to stretch out the soft tissues: tilted to the contralateral side of the release with the chin rotated towards the ipsilateral side. We admit these patients for overnight observation for pain control, and use valium for muscle spasm. The patient remains in the pinless halo in the overcorrected position for 2 weeks at all times followed by night time only for 2 weeks.

## 3. Methods

Investigational review board approval was obtained prior to study initiation. Patients were retrospectively identified who had a diagnosis of CMT, were less than 18 years of age, and underwent bipolar release with partial resection of the SCM muscle from January 1, 2004, to February 14, 2019, with a minimum follow-up of 6 weeks. Patient charts were reviewed for age, sex, surgical technique, prior surgeries, surgical time, complications, reoperations, and clinical exam findings (range of motion, head position, SCM contour, and wound healing). Patients were excluded if they were missing preoperative clinical range of motion scores. Written informed consent was obtained from the patient and legal guardian whose images were included in this case report. Informed consent and Health Insurance Portability and Accountability Act authorization for the remaining patients were otherwise waived by our Institutional Review Board, given the retrospective nature and minimal risk of the study. Statistical significance was defined as *P* < .05. Statistical analysis was performed using STATA/IC 14.0 (Stata Statistical Software:Release 14; StataCorp LP, 2015, College Station, TX). Continuous outcome variables are reported as mean ± standard deviation and were compared preoperatively and at the final follow-up using Student *t* tests.

## 4. Results

Twenty patients underwent a bipolar release with resection of the SCM muscle by the senior author. Nine patients did not have preoperative range of motion recorded and 2 patients did not have 6 weeks of follow-up, leaving 9 patients (6 males, 3 females) to include in the results. Preoperatively all patients had failed a trial of physical therapy and all conservative measures were exhausted. All patients were at least 3 years of age and average age at the time of surgery was 9.3 ± 3.4 years (range: 3.3–13.7 years). The majority of patients had some degree of facial asymmetry noted preoperatively. Six (66.7%%) of the surgeries were on the right SCM muscle and 3 (33.3%) were on the left. There were no surgical complications. The average duration of surgery was 62.2 ± 10.3 minutes (range: 51–74 minutes). The average inpatient stay was 1.2 ± 0.4 days (range: 1–2 days). Postoperatively patients underwent pinless halo full-time use for 2 to 4 weeks, followed by nighttime only use for an additional 2 to 4 weeks. At the point that the pinless halo was transitioned to nighttime use, either physical therapy or a home stretching regimen is initiated. The average duration of final follow-up was 189.6 ± 140.4 days (range: 54–441 days). No patients required subsequent revision surgery. One patient in this series underwent a unipolar release at age 4 after which he resumed physical therapy but was then lost to follow-up. He subsequently underwent a bipolar release with z-plasty at an outside hospital and then represented to our institution and underwent his third SCM release at the age of 12 using the technique described.

Preoperatively, the 6 patients with right SCM tightness presented with their head tilted to the right and rotated to the left, and the 3 patients with left SCM tightness presented with their head tilted to the left and rotated to the right as would be expected. The average preoperative ipsilateral rotational range of motion was 59.7° ± 26.5°. The average contralateral rotational range of motion was 83.8° ± 11.1° (Table 1).

**Table 1:** Average rotational range of motion of each side (°).

	Preoperative	Final	*P* value
Ipsilateral side	59.7 ± 26.5	85.6 ± 7.3	.01
Contralateral side	83.8 ± 11.1	85.6 ± 8.8	.71

At the final follow-up, 8 (88.9%) patients had neutral head positioning. The 1 patient who did not have a neutral head position started with a head tilt to the right prior to surgery and had minimal right head tilt of 5° at the final follow-up (slight under-correction of head tilt). Ipsilateral rotational range of motion significantly improved from preoperatively to final follow-up (59.7° ± 26.5° vs 85.6° ± 7.3°, *P = *.01). Contralateral rotational range of motion was maintained from preoperative to final follow-up (83.8° ± 11.1° vs 85.6° ± 8.8°, *P = *.71) (Table 1). See Table 2 for the range of motion for each patient. All of the patients maintained normal appearing contour of the operative SCM. There were no wound issues although one of the patients developed a small keloid. Data sharing not applicable to this article as no datasets were generated or analyzed during the current study.

**Table 2:** Preoperative and postoperative range of motion exams for each patient (°)

Patient	Preoperative ipsilateral	Preoperative contralateral	Final ipsilateral	Final contralateral
1	70	60	90	90
2	80	90	90	90
3	80	90	80	90
4	60	90	90	90
5	45	90	70	70
6	0	90	90	90
7	80	70	90	90
8	77	84	90	90
9	45	90	80	70

## 5. Discussion

The current case series presents the outcomes of a new technique with bipolar release with partial SCM resection in the pediatric patient population. The findings from our study suggest this is a favorable technique compared to that described in other studies on pediatric patients with improved clinical head position and range of motion with no complications and fewer reoperations (no patients in our cohort required revision surgery). One of the patients had 2 prior procedures including a unipolar release followed by a bipolar release with z-plasty. This contrasts the results by Ferkel et al^[6]^ that did not report any reoperations from their described technique of bipolar release with SCM z-plasty.

Eight of the 9 patients in our cohort achieved neutral head positioning at the final follow-up, with one patient having a mild 5° tilt. This finding was similar in the 8 patients who had a minimum follow-up of 6 weeks but were excluded because they did not have preoperative ROM measurements, who all achieved neutral head positioning at the final follow-up. Ipsilateral rotational range of motion at the final follow-up was significantly improved from that preoperatively, and contralateral rotational range of motion at the final follow-up was maintained. This is in contrast to Ling et al^[17]^ that analyzed outcomes in pediatric patients undergoing SCM release and found that only 33% of patients 5 to 8 years of age, and 0% of patients greater than 9 years of age had favorable outcomes. Ferkel et al^[6]^ also showed poor results in pediatric patients who underwent either a unipolar or bipolar release with 50% having residual postoperative head tilt. Finally, Hollier et al^[10]^ published a 50% rate of reoperation after bipolar SCM release without resection.

There are few studies in the literature that describe the results of SCM release for CMT in patients under 8 years of age. Our study adds to the literature by including a patient cohort with an average age of 9.3 years (range 3–13 years of age).^[7,15,16]^ This may be relevant as Shim et al^[7]^ found that patients who were done growing (which they defined as 15–24 years old) had worse improvement following unipolar or bipolar release compared to those patients who were still growing (patients 8–14 years old). Our study also found favorable results in a relatively young patient population undergoing bipolar SCM with partial resection. Our sample size is too small to draw any significant conclusions as to an ideal age when this surgery should be performed. Similar to most procedures, it is a joint decision between the family and surgeon while taking into consideration the overall health and size of the patient as well as the magnitude and progression of the deformity.

Unlike other techniques for treating CMT, which involve incisions as large as 5 cm, our technique utilizes a smaller incision of 2 to 3 cm. Bipolar release with z-plasty requires greater SCM exposure to cut and lengthen the muscle, while release with partial resection requires minimal exposure of the SCM. Consequently, the smaller incision associated with the partial resection technique minimizes visible surgical scarring while improving overall cosmesis.

Interestingly, the distribution of right and left CMT (66.7% right, 33.3% left) is very similar to that of Shim et al,^[7]^ who had 73.7% right and 26.3% left in their cohort age 8 to 14 years. Although it is unclear why the right side was affected more than the left in our study, Petronic et al^[4]^ found a higher rate of right-sided CMT, independent of age or sex. Further studies on the pathophysiology of CMT are needed to further clarify this finding.

Due to the retrospective nature of this study, there are inherent limitations. Data collection was limited to what was available in the patient notes and some of the initial cohort was excluded due to the lack of preoperative clinical range of motion and/or head tilt examinations recorded. As a result, the sample size of the study was limited, which was also due to the rarity of the condition. However, our cohort was still comparable to that of the other published studies. Our period of follow-up does leave open the possibility that our results underestimate the incidence of longer-term complications such as recurrence or reoperation. Nevertheless, our cohort included multiple patients with 6 months of follow-up as well as a couple with 14 months, all of whom had similar outcomes postoperatively to the patients with only 6 weeks of follow-up. It is also worth noting that as our technique represents a more aggressive resection than what has been previously described, the biggest concern with this modification would not be recurrence that might be present with longer-term follow-up, as this, in theory, should help guard against that. Rather, the concerns would be creating a defect or overcorrection, both of which one would anticipate presenting early on. Finally, our minimum final follow-up was 6 weeks. This is due to the fact that most of these patients recover quickly after this relatively minimally invasive procedure and do not require further follow-up. A prospective study with longer follow-up may be helpful in determining the long-term effectiveness of this procedure.

This case series is the first to present the effectiveness and safety of bipolar release with partial SCM resection in the pediatric population. Based on the clinical improvement and lack of complications with no subsequent revision surgery, we feel that it is a viable option for treating CMT in the pediatric population. Further studies with longer follow-up are indicated to confirm the longevity of these initial promising results.

## Author contributions

**Data curation:** Kyle K. Obana, Annika Y. Myers, James T. Bennett, Ali A. Siddiqui, Bensen B. Fan, Lindsay M. Andras, David L. Skaggs.

**Formal analysis:** Kyle K. Obana, Lindsay M. Andras, Tishya A.L. Wren, David L. Skaggs.

**Investigation:** Kyle K. Obana, Annika Y. Myers, James T. Bennett, Ali A. Siddiqui, Bensen B. Fan, Lindsay M. Andras, Tishya A.L. Wren, David L. Skaggs.

**Methodology:** Kyle K. Obana, Annika Y. Myers, James T. Bennett, Ali A. Siddiqui, Bensen B. Fan, Lindsay M. Andras, Tishya A.L. Wren, David L. Skaggs.

**Project administration:** Kyle K. Obana, Annika Y. Myers, James T. Bennett, Ali A. Siddiqui, Bensen B. Fan, Lindsay M. Andras, Tishya A.L. Wren, David L. Skaggs.

**Resources:** Kyle K. Obana, Annika Y. Myers, James T. Bennett, Ali A. Siddiqui, Bensen B. Fan, Lindsay M. Andras, Tishya A.L. Wren, David L. Skaggs.

**Software:** Kyle K. Obana, Tishya A.L. Wren.

**Supervision:** Kyle K. Obana, Lindsay M. Andras, Tishya A.L. Wren, David L. Skaggs.

**Validation:** Kyle K. Obana, Annika Y. Myers, James T. Bennett, Ali A. Siddiqui, Bensen B. Fan, Lindsay M. Andras, Tishya A.L. Wren, David L. Skaggs.

**Visualization:** Kyle K. Obana, Lindsay M. Andras, David L. Skaggs.

**Writing—original draft:** Kyle K. Obana, Lindsay M. Andras, David L. Skaggs.

**Writing—review & editing:** Kyle K. Obana, Annika Y. Myers, Lindsay M. Andras, Tishya A.L. Wren, David L. Skaggs.

**Conceptualization:** Lindsay M. Andras, David L. Skaggs.
